# PTSD-Related Behavioral Traits in a Rat Model of Blast-Induced mTBI Are Reversed by the mGluR2/3 Receptor Antagonist BCI-838

**DOI:** 10.1523/ENEURO.0357-17.2018

**Published:** 2018-01-30

**Authors:** Georgina Perez-Garcia, Rita De Gasperi, Miguel A. Gama Sosa, Gissel M. Perez, Alena Otero-Pagan, Anna Tschiffely, Richard M. McCarron, Stephen T. Ahlers, Gregory A. Elder, Sam Gandy

**Affiliations:** 1 Research and Development, James J. Peters Veterans Affairs Medical Center, Bronx, NY 10468; 2 Department of Neurology and NFL Neurological Care Center, Icahn School of Medicine at Mount Sinai, New York, NY 10029; 3 Department of Psychiatry and Alzheimer’s Disease Research Center, Icahn School of Medicine at Mount Sinai, New York, NY 10029; 4 Department of Neurotrauma, Operational and Undersea Medicine Directorate, Naval Medical Research Center, Silver Spring, MD 20910; 5 Department of Surgery, Uniformed Services University of the Health Sciences, Bethesda, MD 20814

**Keywords:** BCI-838, blast, metabotropic glutamate receptor, mGluR2/3, posttraumatic stress disorder, traumatic brain injury

## Abstract

Battlefield blast exposure related to improvised explosive devices (IEDs) has become the most common cause of traumatic brain injury (TBI) in the recent conflicts in Iraq and Afghanistan. Mental health problems are common after TBI. A striking feature in the most recent veterans has been the frequency with which mild TBI (mTBI) and posttraumatic stress disorder (PTSD) have appeared together, in contrast to the classical situations in which the presence of mTBI has excluded the diagnosis of PTSD. However, treatment of PTSD-related symptoms that follow blast injury has become a significant problem. BCI-838 (MGS0210) is a Group II metabotropic glutamate receptor (mGluR2/3) antagonist prodrug, and its active metabolite BCI-632 (MGS0039) has proneurogenic, procognitive, and antidepressant activities in animal models. In humans, BCI-838 is currently in clinical trials for refractory depression and suicidality. The aim of the current study was to determine whether BCI-838 could modify the anxiety response and reverse PTSD-related behaviors in rats exposed to a series of low-level blast exposures designed to mimic a human mTBI or subclinical blast exposure. BCI-838 treatment reversed PTSD-related behavioral traits improving anxiety and fear-related behaviors as well as long-term recognition memory. Treatment with BCI-838 also increased neurogenesis in the dentate gyrus (DG) of blast-exposed rats. The safety profile of BCI-838 together with the therapeutic activities reported here, make BCI-838 a promising drug for the treatment of former battlefield Warfighters suffering from PTSD-related symptoms following blast-induced mTBI.

## Visual Abstract

**Figure:**
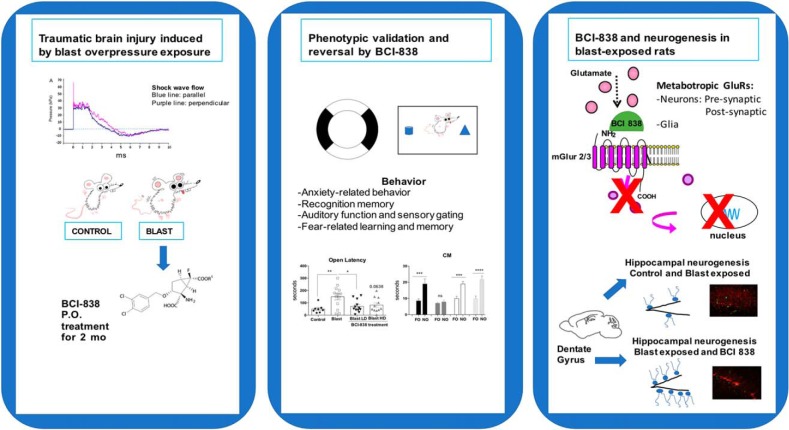

## Significance Statement

Currently available therapies are only partially effective for the treatment of posttraumatic stress disorder (PTSD)-related symptoms that appear following blast injury. Treatment with the proneurogenic mGluR2/3 receptor antagonist BCI-838 reversed PTSD-related behavioral traits in a rat model of blast-related mild traumatic brain injury (mTBI). This study highlights BCI-838/BCI-632 and the mGluR2/3 pathway as potential leads in development of novel pharmacological therapies for PTSD-related symptoms that follow blast injury.

## Introduction

Traumatic brain injury (TBI) is a major cause of combat-related disability (Gubata et al., 2014; Elder, 2015). Military-related TBIs occur through various mechanisms. Because of the widespread use of improvised explosive devices (IEDs) in Iraq and Afghanistan blast-related mechanisms have been the most common cause (Hoge et al., 2008; Tanielian and Jaycox, 2008). Further, as survival after battlefield trauma has improved, TBI has become recognized as a particularly common injury in the recent conflicts in Iraq and Afghanistan with estimates that as many as 20% of returning veterans suffered a TBI during deployment (Hoge et al., 2008; Tanielian and Jaycox, 2008). Initially, most attention focused on the moderate to severe end of the injury spectrum, which was the type of TBI that would be recognized acutely in theater. However, what soon became clear was that most TBIs being suffered in these conflicts were mild TBI (mTBI) with many going undocumented at the time of occurrence (Chase, 2015).

Mental health problems occur often after TBI (Jorge et al., 2014). Indeed, a striking feature in the most recent veterans has been the frequency with which posttraumatic stress disorder (PTSD) has been seen following blast-related mTBI (Elder et al., 2010). Studies in Iraq veterans have found that over one-third suspected of having an mTBI-related postconcussion syndrome also have PTSD or depression (Hoge et al., 2008; Vasterling et al., 2009). This dual diagnosis of PTSD and mTBI upends the conventional diagnostic separation of the two entities. Indeed, the presence of mTBI has traditionally excluded the diagnosis of PTSD. While controversy remains over the separation of the two disorders clinically (Hoge et al., 2009; Bryant, 2011; Elder et al., 2014) there is no doubt that mental health problems are common following blast-related mTBI.

BCI-838 (MGS0210), bicycle [3.1.0] hexane-2,6-dicarboxylic acid, 2-amino-3-[(3,4-dichlorophenyl)metoxy]-6-fluoro-,6-heptyl ester,(IR,2R,3R, 5R6R)-), is the prodrug for BCI-632 (MGS0039), a Group II metabotropic glutamate receptor (mGluR2/3) antagonist. BCI-838 has been found to improve memory and reduce anxiety in an animal model of Alzheimer’s disease (Kim et al., 2014). It has been tested in humans and found to be clinically well tolerated and orally bioavailable. BCI-838 is currently in human clinical trials for depression. As a prodrug BCI-838 is metabolized in the liver into BCI-632, which is the active compound delivered to brain. In humans, daily oral dosing of BCI-838 results in steady-state levels in brain, which last for 22 h. mGluR2/3 receptor antagonists are proneurogenic as evidenced by their stimulation of hippocampal neurogenesis in adult brain (Yoshimizu and Chaki, 2004). In rodents, mGluR2/3 receptor antagonists enhance learning and memory and they also possess anxiolytic and antidepressive properties (Higgins et al., 2004; Shimazaki et al., 2004; Yoshimizu et al., 2006; Campo et al., 2011). As a class, these compounds are regarded as promising for treatment of a variety of mental health and neurologic disorders including refractory major depression, suicidality, sleep-wake cycle disorders, and other psychiatric conditions in which cognitive impairment is a prominent feature (Celanire et al., 2015).

Neurogenesis in the adult hippocampus affects higher cognitive functions (especially memory) and influences affective behavior (Kempermann et al., 2015). Stimulation of hippocampal neurogenesis has been proposed as a central mechanism underlying the action of antidepressant drugs (Chaki, 2017). TBI by cortical impact is reported to impair hippocampal neurogenesis (Rola et al., 2006; Shetty, 2014; Wang et al., 2016; Shapiro, 2017), raising the possibility that proneurogenic drugs might be effective in modifying the course of latent manifestations resulting from mTBI.

Modulation of other mGluRs has also been explored in experimental models of TBI (Loane et al., 2009, 2013; Kabadi and Faden, 2014). The stimulation of mGluR5 has emerged as one of the more promising approaches with effects that include promoting reduced production of nitric oxide and tumor necrosis factor-α as well as limiting caspase dependent apoptosis and intracellular generation of reactive oxygen species (Loane et al., 2009). However, none of these studies have explored modulation of mGluRs in the context of blast injury.

The aim of this study was to investigate whether administration of BCI-838 could modify the anxiety response and reverse PTSD-related behaviors while concomitantly enhancing neurogenesis in the dentate gyrus (DG) in rats previously found to exhibit a variety of chronic PTSD-related behavioral traits (Elder et al., 2012; Perez-Garcia et al., 2016, 2018). BCI-838 treatment reversed PTSD-related traits improving anxiety and fear-related behaviors, in addition to long-term recognition memory. Hippocampal neurogenesis was also robustly increased in the DG of drug-treated blast-exposed rats. The present study highlights the potential role for BCI-838, hippocampal neurogenesis, and the mGluR2/3 pathway in the development of novel pharmacological therapies to help former Warfighters suffering from the dual diagnosis status when PTSD-related symptoms coexist with blast-induced mTBI.

## Materials and Methods

### Animals

Adult male Long Evans Hooded rats (250–350 g; 10–12 weeks of age; Charles River Laboratories International, Inc.) were used as subjects. All studies were approved by the Institutional Animal Care and Use Committees of the James J. Peters VA Medical Center and the Walter Reed Army Institute of Research/Naval Medical Research Center. Studies were conducted in compliance with the Public Health Service policy on the humane care and use of laboratory animals, the NIH Guide for the Care and Use of Laboratory Animals, and all applicable Federal regulations governing the protection of animals in research.

### Blast overpressure exposure

Rats were exposed to overpressure injury using a shock tube, which simulates the effects of air blast exposure under experimental conditions (Ahlers et al., 2012). The shock tube has a 0.32-m circular diameter and is a 5.94 m-long steel tube divided into a 0.76-m compression chamber that is separated from a 5.18-m expansion chamber. The compression and expansion chambers are separated by polyethylene terephthalate Mylar TM sheets (Du Pont Co) that control the peak pressure generated. The peak pressure at the end of the expansion chamber was determined with piezoresistive gauges specifically designed for pressure-time (impulse) measurements (Model 102M152, PCB, Piezotronics, Inc.).

Individual rats were anesthetized using an isoflurane gas anesthesia system consisting of a vaporizer, gas lines and valves and an activated charcoal scavenging system adapted for use with rodents. Rats were placed into a polycarbonate induction chamber, which was closed and immediately flushed with 5% isoflurane mixture in air for two minutes. Rats were placed into a cone shaped plastic restraint device and then placed in the shock tube. Movement was further restricted during the blast exposure using 1.5 cm in diameter flattened rubber tourniquet tubing. Three tourniquets were spaced evenly to secure the head region, the upper torso and lower torso while the animal was in the plastic restraint cone. The end of each tubing was threaded through a toggle and run outside of the exposure cage where it was tied to firmly affix the animal and prevent movement during the blast overpressure exposure without restricting breathing. Rats were randomly assigned to sham or blast conditions with the head facing the blast exposure without any body shielding resulting in a full body exposure to the blast wave. The total length of time under anesthesia including placement in the shock tube and execution of the blast procedure was typically <3 min. Blast-exposed animals received 74.5 kilopascal (kPa) exposures equivalent to 10.8 pounds per square inch (psi). One exposure per day was administered for three consecutive days. Sham exposed animals were treated identically including receiving anesthesia and being placed in the blast tube but did not receive a blast exposure. Under the blast conditions used here blast-exposed rats recovered identically to controls and exhibited no loss of the righting reflex (Ahlers et al., 2012).

### Animal housing

Animals were housed at a constant 70-72°F temperature with rooms on a 12/12 h light/dark cycle with lights on at 7 A.M. All subjects were individually housed in standard clear plastic cages equipped with Bed-O'Cobs laboratory animal bedding (The Andersons) and EnviroDri nesting paper (Sheppard Specialty Papers). Access to food and water was ad libitum. Subjects were housed on racks in random order to prevent rack position effects. Cages were coded to allow maintenance of blinding to groups during behavioral testing.

### Drug administration

BCI-838 was dissolved in a solution of 5% carboxymethylcellulose (CMC; Sigma Aldrich) and 0.3% 2 N hydrochloric acid solution (Sigma Aldrich) at room temperature. The drug emulsion was prepared daily by sonication for 2 min to fully dissolve. Animals were divided into four experimental groups: (1) sham exposed (placed in blast tube but did not receive blast exposure) treated with vehicle (5% CMC); (2) blast exposed treated with vehicle; (3) blast exposed treated with 4-mg/kg BCI-838 (low dose); and (4) blast exposed treated with 10-mg/kg BCI-838 (high dose). The experiment was performed independently on two cohorts of rats described in Extended Data Figures 1-1, 1-2. Doses were chosen based on previous work in other rodent models (Kim et al., 2014). Bodyweight was recorded weekly and doses were adjusted accordingly.

**Figure 1.**
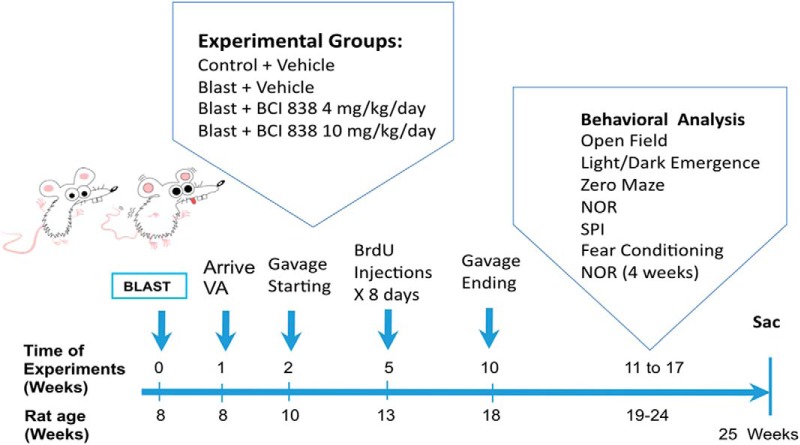
Experimental design and timing. Further description of both cohorts is contained in Extended Data Figures 1-1, 1-2.

10.1523/ENEURO.0357-17.2018.f1-1 Extended Data Figure 1-1 Details of statistical analysis cohort one. Download Figure 1-1, DOCX file.

**Figure 2.**
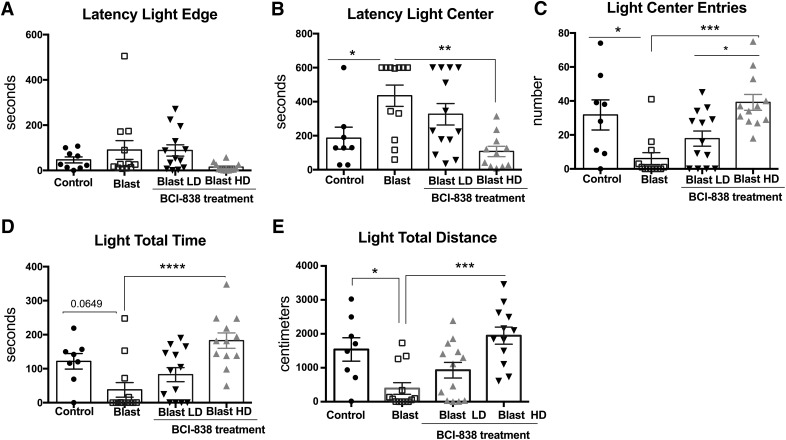
High-dose BCI-838 reverses anxiety in the light/dark emergence task. While the light edge latency (***A***) was unchanged, light center latency (***B***) was increased in blast-exposed rats, which also made fewer entries (***C***), and traveled less distance on the light side (***E***). Treatment with high-dose BCI-838 (10 mg/kg) reversed these effects. Values significantly different from controls are indicated by asterisks (*p < 0.05, **p < 0.01, ***p < 0.001, and ****p < 0.0001, Tukey’s or Sidak’s multiple comparisons tests). Values are expressed as mean ± SEM. Results from cohort one can be found in Extended Data Figure 2-1.

10.1523/ENEURO.0357-17.2018.f1-2 Extended Data Figure 1-2 Details of statistical analysis cohort two. Download Figure 1-2, DOCX file.

The drug was administered by oral gavage starting two weeks after the last blast exposure. Administration was conducted daily between 9 A.M. and 2 P.M. for 60 d by personnel experienced in the procedure. Restraint for gavage was performed similar to that described by Turner et al. (2012) except that a towel was used to firmly grasp and gently immobilize the rat with the head and body held vertically. A 7-cm straight stainless-steel gavage needle with a 3-mm ball tip (Fischer Scientific) was used for gavage and wiped clean between animals.

### Bromodeoxyuridine (BrdU) injections

All animals received once daily intraperitoneally injections of BrdU (150 mg/kg of body weight) for 8 d during the third week of drug treatment (five weeks after blast exposure). BrdU (Sigma) was dissolved in saline solution (0.9% NaCl in sterile H_2_O) warmed to 40°C and gently vortexed. The solution was allowed to cool to room temperature (25°C) before injection.

### Behavioral testing

Behavioral testing was begun at the end of the 60 d of drug administration. All behavioral testing was performed by the same investigator (GPG). The following tests were performed.

#### Locomotor activity and open field

General locomotor activity and open field behavior was examined in 40.6 × 40.6 cm Versamax activity cages (Accuscan), each outfitted with a grid of 32 infrared beams at ground level and 16 elevated 7.6 cm above ground level. Locomotor activity was recorded during 60 min and analyzed with VersaData Software (Accuscan), which automatically calculates move time, move distance and center time based on beam breaks. The center of the chamber was defined as a square of 25.4 × 25.4 cm (7.6 cm from each side wall) and virtually drawn with VersaMap software (Accuscan). Center entries and center rest time were defined based on the centroid of the rat being in the center of the chamber with center rest time defined as time when the centroid was in the center of the chamber but during which no beam breaks were generated. Samples were recorded in 1-min bins and summed into 5-min intervals for presentation.

#### Light/dark emergence

A light/dark emergence task was run in Versamax activity cages with opaque black Plexiglas boxes enclosing the left half of the interiors so that only the right sides were illuminated. Animals began in the dark side and were allowed to freely explore for 10 min with access to the left (light) side through an open doorway located in the center of the monitor. Subject side preference and emergence latencies were tracked by centroid location with all movement automatically tracked and quantified. Light-side emergence latency, time to reach the center of the lighted side (light side center latency) and percentage total light-side duration were calculated from beam breaks. All equipment was wiped clean between tests.

#### Elevated zero maze (EZM)

The apparatus consisted of a circular black Plexiglas runway 121.92 cm in diameter and raised 76 cm off the floor (San Diego Instruments). The textured runway itself was 5.08 cm across and divided equally into alternating quadrants of open runway enclosed only by a 1.27-cm lip and closed runway with smooth 15.24-cm walls. All subjects received a 5-min trial beginning in a closed arc of the runway. During each trial, subjects were allowed to move freely around the runway, with all movement tracked automatically by a video camera placed on the ceiling directly above the maze. Data were analyzed by ANYMAZE (San Diego Instruments) yielding measures of total movement time and distance for the entire maze, as well as time spent and distance traveled in each of the individual quadrants. From the quadrant data, measures of total open and closed arc times, latency to enter an open arc, total open arm entries and latency to completely cross an open arc between two closed arcs were calculated. Subject position was determined by centroid location.

#### Novel object (NO) recognition

Rats were habituated to the arena (90 cm length × 60 cm width × 40 cm height) for 20 min, 24 h before training. On the training day, two identical objects were placed on opposite ends of the empty arena, and the rat was allowed to freely explore the objects for 7 min. After a 1-h delay, during which the rat was held in its home cage, one of the two familiar objects (FOs) was replaced with a novel one, and the rat was allowed to freely explore the familiar and NO for 5 min to assess short-term memory (STM). After a 24-h delay, during which the rat was held in its home cage, one of the two FOs was replaced with a novel one different from the ones used during the STM test. The rat was allowed to freely explore the familiar and NO for 5 min to assess long-term memory (LTM). After a four-week delay (from training), during which the rat was held in its home cage, one of the two FOs used during the LTM testing was replaced with a novel one different from those used during either the STM or LTM tests. The rat was allowed to freely explore the familiar and NO for 5 min to assess consolidation memory (CM). Raw exploration times for each object were expressed in seconds. Object exploration was defined as sniffing or touching the object with the vibrissae or when the animal’s head was oriented toward the object with the nose placed at a distance of <2 cm from the object. All sessions were recorded by video camera (Sentech) and analyzed with ANYMAZE software (San Diego Instruments). In addition, offline analysis by an investigator blind to the blast-exposed status of the animals was performed. Objects to be discriminated were of different size, shape and color and were made of plastic or metal material. The objects consisted of a 330-ml soda can, a metal box, a cup and a plastic tube. All objects were cleaned with 70% ethanol between trials.

#### Prepulse inhibition (PPI) and acoustic startle

Startle magnitude and sensory gating were examined in a 40-trial PPI assay (San Diego Instruments). Animals were placed in isolation chambers inside closed Plexiglas tubes, each of which was mounted on a platform resting on an accelerometer. Following a 5-min habituation period with 74-dB background white noise, each animal received 40 randomized trials separated by 20-30 s. Trials consisted of 10 each of background readings taken at 74 dB, startle trials with readings following 40-ms 125-dB tones, PPI trials where the 125-dB tone was preceded 100 ms earlier by a 20-ms 79-dB tone and control trials consisting of only the 20-ms 79-dB prepulse. On all trials, maximum magnitude of the animal’s startle (or other motion) was automatically recorded in 500-ms windows by an accelerometer. The tubes were rinsed clean between animals. Percentage PPI was calculated with the formula 100 – (startle response on acoustic prepulse plus pulse stimulus trials/pulse stimulus response alone trials) × 100. The first startle response was compared among groups.

#### Contextual and cued fear conditioning

Sound-attenuated isolation cubicles (Coulbourn Instruments) were used. Each cubicle was equipped with a grid floor for delivery of the unconditioned stimulus (US) and overhead cameras. All aspects of the test were controlled and monitored by the Freeze Frame conditioning and video tracking system (Actimetrics, Coulbourn Instruments). During training the chambers were scented with almond extract, lined with white paper towels, had background noise generated by a small fan and were cleaned before and between trials with 70% ethanol. The tester wore latex gloves. Each subject was placed inside the conditioning chamber for 2 min before the onset of a conditioned stimulus (CS; an 80 dB, 2-kHz tone), which lasted for 20 s with a coterminating 2-s footshock (0.7 mA; US). Each rat remained in the chamber for an additional 40 s following the CS-US pairing before being returned to its home cage. Freezing was defined as a lack of movement (except for respiration) in each 10-s interval. Minutes 0–2 during the training session were used to measure baseline freezing. Contextual fear memory testing was performed 24 h after the training session by measuring freezing behavior during a 3-min test in the conditioning chamber under conditions identical to those of the training session with the exception that no footshock or tone (CS or US) was presented. Animals were returned to their home cage for another 24 h, at which time cued conditioning was tested. To create a new context with different properties, the chambers were free of background noise (fan turned off), lined with blue paper towels, scented with lemon extract and cleaned before and during all trials with isopropanol. In addition, the tester wore nitrile gloves and habituated the rats pretesting in a different holding room. Each subject was placed in this novel context for 2 min and baseline freezing was measured, followed by exposure to the CS (20-s tone) at 120 and 290 s.

### Tissue processing and immunohistochemistry

Animals were sacrificed at the conclusion of behavioral testing. After deep anesthesia with a solution of 150 mg/kg ketamine and 30 mg/kg xylazine, rats were euthanized by transcardial perfusion with cold 4% paraformaldehyde in PBS. After perfusion, brains were removed and postfixed in 4% paraformaldehyde for 48 h, transferred to PBS, and stored at 4°C until sectioning. Fifty-micrometer-thick coronal sections were cut through the entire extend of the hippocampus using a Leica VT1000 S Vibratome (Leica). The sections were stored at −20°C in a cryoprotectant solution (25% ethylene glycol and 25% glycerine in 0.05 M PBS) until processing for immunofluorescence.

For stereologically based counting every 6th section in a series was processed for immunohistochemistry so that the interval between sections within a given series was 300 μm. For BrdU staining, the sections from each brain were treated with 50% formamide and 2 × SSC (0.3 M NaCl and 0.03 M sodium citrate) for 2 h, followed by incubation with 0.1 M boric acid buffer at pH 8.5 for 10 min. After 4 × 5 min washes with PBS, they were incubated in blocking buffer (3% goat serum, 0.3% Triton X-100 in PBS) for 1 h and incubated overnight at 4°C in a mixture of rat anti-BrdU (1:300, Abcam) plus rabbit anti-neuron-specific nuclear protein (NeuN, 1:500; Millipore) antibodies. The next day, sections were washed 4 × 5 min with PBS and exposed for 2 h in the dark with Alexa Fluor 568-conjugated donkey anti-rat IgG and with Alexa Fluor 488-conjugated goat anti-rabbit IgG (Life Technologies). Both secondary antibodies were used at a dilution of 1:300. To ascertain the effects of BCI-838 on cell proliferation and survival, a second series of sections from each animal was immunolabeled with doublecortin (DCX) and BrdU as described above using a goat monoclonal anti-DCX antibody (1:500 from Santa Cruz). All slices were mounted onto slides and covered under Fluoro-Gel (with Tris Buffer from Electron Microscopy Sciences).

### Image analysis and neurogenesis quantification

Given the scarcity of BrdU- and DCX-immunostained cells, the number of new cells was estimated using a modified version of the optical fractionator method employing an exhaustive sampling scheme. All BrdU- or DCX-labeled cells were counted on both sides of every 6th bilateral section throughout the entire DG between coordinates −2.52 and 5.40 mm relative to bregma. Immunostained cells were first visualized with a 40× objective. To ensure accurate comparison between groups, we checked that section thicknesses were similar for all the groups with the aid of a microcator focused on immuno-fluorescence labeled nuclei at the border of the hilus and DG. The number of BrdU- or DCX-labeled cells per granule cell layer (GCL, including the SGZ) was estimated using the following formula: N = Q × (1/ssf), where Q is the total number of counted cells and 1/ssf is the reciprocal of the section sampling fraction (1/ssf = 12 in the present case).

For quantification of double-labeled BrdU/NeuN cells, 11 bilateral slices per animal spanning the entire DG were used to determine the frequency of BrdU-positive cells expressing NeuN. Eight to 12 optical sections (1 μm thick) were scanned from each area using the 40× objective. BrdU-labeled cells were scored as neurons when the NeuN labeling was unambiguously associated with a BrdU-positive nucleus in the stack of sections. The percentages of BrdU-labeled cells that were also labeled with NeuN were calculated for each group.

### Statistical analysis

Values are expressed as mean ± SEM. Extended Data Figures 1-1, 1-2 contain the data structure, type of test used, observed power, and *n* for each figure. Each test included enough animals to reach a power close to or higher than 0.8. Statistical tests were performed using the program GraphPad Prism 7.0 (GraphPad Software), IBM SPSS statistics 24, and G* Power (Heinrich-Heine-Universität Düsseldorf). To systematically test for normality the D’Agostino–Pearson and Shapiro Wilk tests were used. Depending on the behavioral test, multiple comparisons were performed using one-way ANOVA for normally distributed datasets followed by Tukey’s *post hoc* tests for multiple comparisons when appropriate. The datasets used for two-way repeated measures ANOVA were normally distributed and were followed by *post hoc* Sidak’s test.

## Results

### Treatment of blast-exposed rats with BCI-838

We studied a model of blast exposure using rats. Because multiple blast exposures have been common among former Warfighters returning from Iraq and Afghanistan (Hoge et al., 2008; Tanielian and Jaycox, 2008; Elder et al., 2010), we used a design in which rats received three 74.5-kPa exposures delivered once per day on three consecutive days. Studies using this model have established that exposures up to 74.5 kPa (equivalent to 10.8 psi), while representing a level of blast that is transmitted to brain, produce no gross neuropathological effects, and histologic examination of the lungs show no hemorrhage or other pathology (Ahlers et al., 2012; Elder et al., 2012). Based on our experience with this model, we believe that these blast pressures mimic a low-level blast exposure equivalent to a human mTBI or subclinical blast exposure.

**Figure 3.**
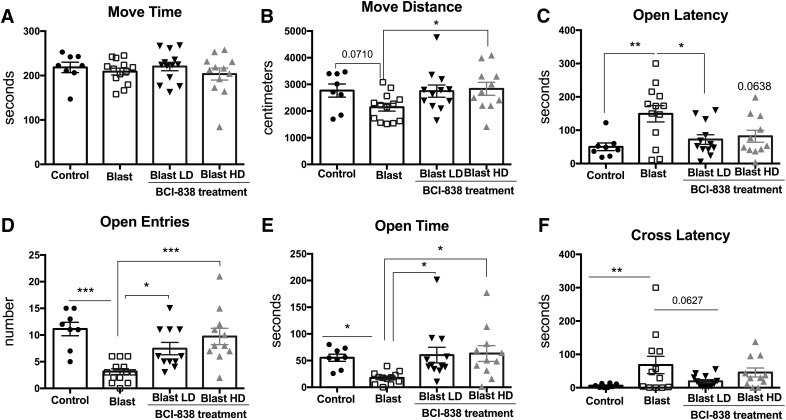
Blast-induced anxiety is reversed in an EZM by BCI-838. While time in motion (***A***) did not differ, blast-exposed rats moved less (***B***) exhibited a longer latency to enter an open arm (***C***), made fewer open arm entries (***D***) as well as spent less time in the open arms (***E***) and exhibited an increased latency to cross between two open arms (cross latency; ***F***). Treatment with both low- and high-dose BCI-838 reversed nearly all of these effects. Values significantly different between controls and blast-exposed rats are indicated by asterisks (*p < 0.05, **p < 0.01, ***p < 0.001, Tukey’s or Sidak’s multiple comparisons tests). Values are expressed as mean ± SEM.

**Figure 4.**
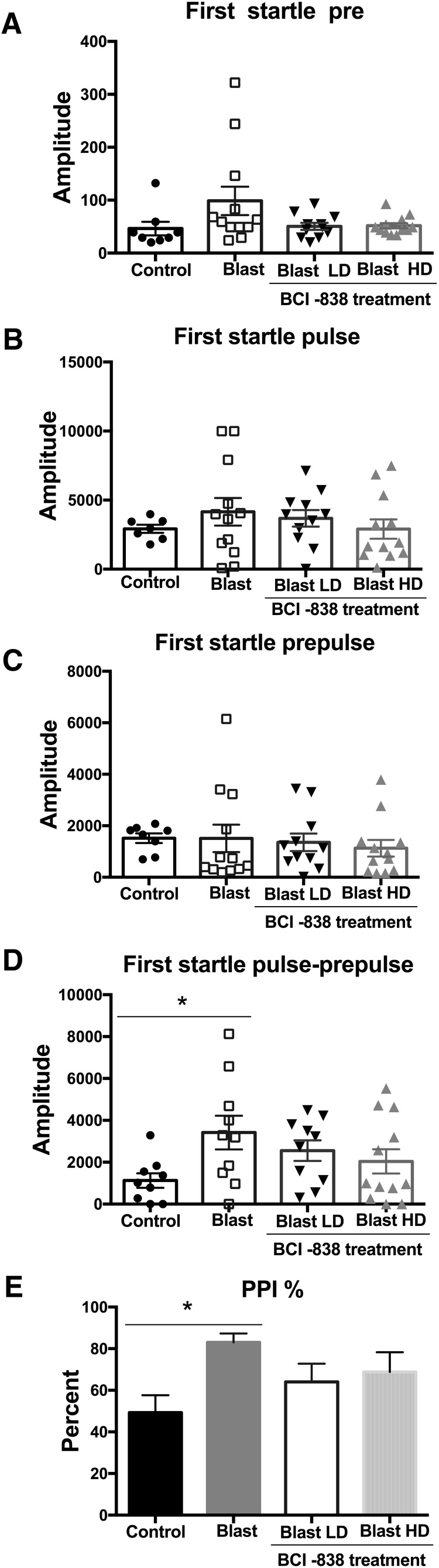
BCI-838 treatment does not rescue enhanced PPI found in blast-exposed rats. Startle magnitude and sensory gating were examined in a PPI assay. We analyzed acoustic startle and PPI of the first startle response. No differences were found in background readings (pre; ***A***), acoustic startle response (pulse; ***B***), or startle following the prepulse (prepulse; ***C***), but we found an increased response when the prepulse was subtracted from the pulse (pulse-prepulse; ***D***) and in the percentage PPI in blast-exposed rats versus the control group (***E***). Treatment with BCI-838 did not normalize either of these responses. Values significantly different between controls and blast-exposed rats are indicated by asterisks (*p < 0.05, Tukey’s multiple comparisons test). Values are expressed as mean ± SEM in all panels.

**Figure 5.**
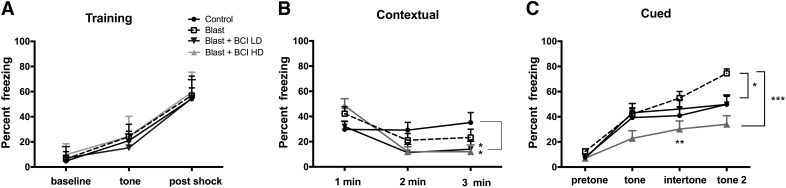
High-dose BCI-838 reverses altered cued fear responses in blast-exposed rats. ***A***, During the training phase, freezing behavior was measured during minutes 0–2 of the training session (baseline), after the presentation of the tone and after the footshock. All groups showed freezing and no differences were found among groups. ***B***, The test for contextual fear memory was performed at 24 h in the same conditioning chamber. No differences were found between blast-exposed rats and non-blast-exposed controls. Blast-exposed rats treated with drug showed less freezing compared to non-blast-exposed controls. ***C***, Cued fear memory was tested another 24 h later. Blast-exposed rats showed increased freezing compared with non-blast-exposed controls after the second tone. Blast-exposed rats treated with high drug doses showed less freezing compared with blast exposed treated with vehicle and comparable freezing to non-blast-exposed controls. Asterisks indicate statistically significant differences (*p < 0.05, **p < 0.01, ***p < 0.001, one-way ANOVA, Tukey’s and Sidak’s multiple comparisons test). Values are expressed as percentage ± SEM. Results from cohort one can be found in Extended Data Figure 5-1.

**Figure 6.**
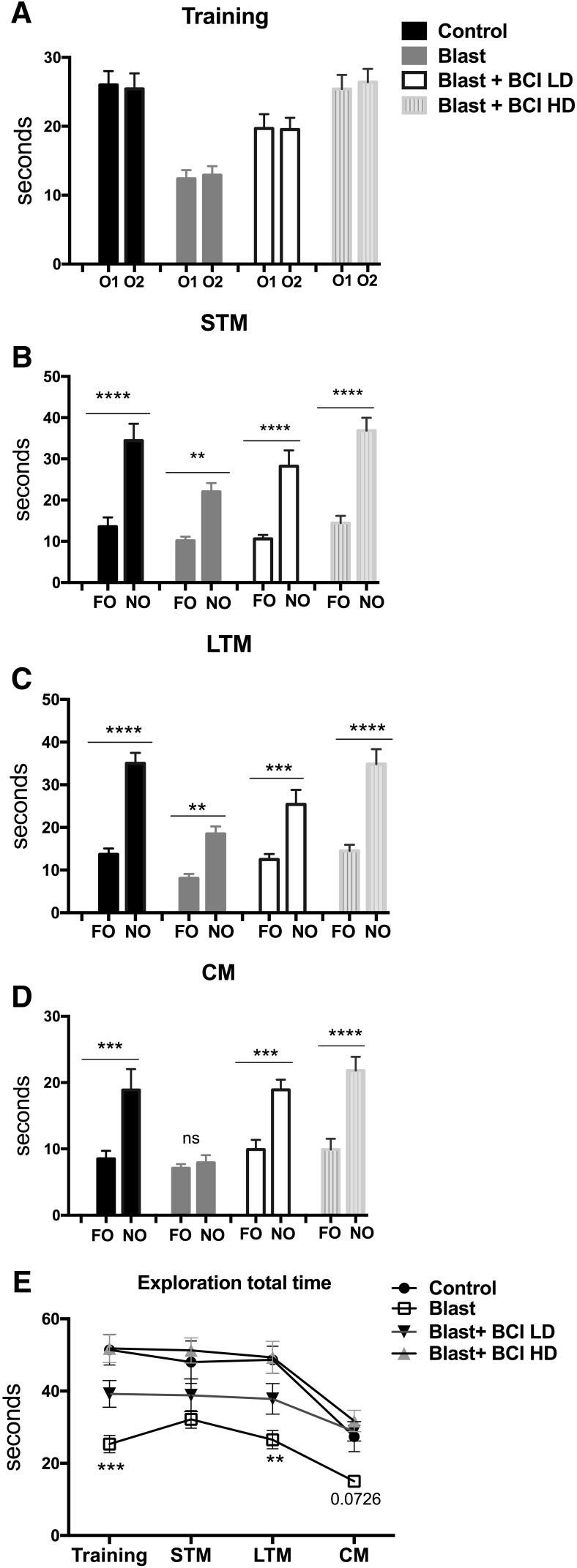
Reduced exploration time as well as late effects on recognition memory in a NOR test were reversed by BCI-838. Blast-exposed rats that were not treated with drug spent less time than controls exploring the objects during the training session (***A***). All groups showed a preference for the NO compared with the FO when tested 1 h (***B***) or 24 h (***C***) later, suggesting that blast does not affect STM or LTM in rats at five months of age. At four weeks after training (***D***), blast-exposed rats showed impaired CM when exploring a FO and NO. This effect was reversed by low dose and high dose of BCI-838. The other groups showed a preference for the NO compared with the FO. Blast-exposed rats generally spent less time exploring the objects than non-blast-exposed controls, an effect that was reversed by both doses of BCI-838 (***E***). Values significantly different from controls and blast-exposed are indicated by asterisks (**p <0.01, ***p < 0.001, ****p < 0.0001, unpaired *t* tests, Student’s in ***A–D***; Tukey’s multiple comparison's test in ***E***). Values are expressed as mean ± SEM.

Since BCI-838 has a variety of potentially relevant neuropsychiatric activities in other rodent models (Kim et al., 2014), we assessed its efficacy in modifying the behavioral traits that follow blast injury. The time course of the experiments is shown in Figure 1. BCI-838 was administered at two different doses (4 and 10 mg/kg/d) by oral gavage starting two weeks after the last blast exposure and was continued for eight weeks. During the third week of drug treatment, BrdU was administered daily for eight consecutive days. Gavage was stopped after week 10 post-blast and behavioral testing was performed between 11 and 17 weeks after blast exposure. Rats were sacrificed at the end of behavioral testing when the animals were 25 weeks old. The experiments were conducted on two cohorts of animals. Results from cohort 2 are primarily described below and presented in Figures 2–6. The effect of BCI-838 treatment in cohort 1 is discussed below and presented in Extended Data Figures 2-1, 5-1. Extended Data Figures 1-1, 1-2, summarize effects in both cohorts.

10.1523/ENEURO.0357-17.2018.f2-1 Extended Data Figure 2-1 Summary of results of the first cohort. High-dose BCI-838 reverses anxiety. In the light/dark emergence task (***A***), the blast-exposed rats exhibited an increased latency to reach the light center, made fewer light center entries, traveled less distance, and spent less time on the light side compared to vehicle-treated controls, effects that were mostly reversed by high-dose BCI-838. In the zero maze (***B***), blast-exposed rats showed an increased open arm cross latency, tended to make fewer open entries, and spent less time in the open arms. These parameters were reversed by BCI-838 (10 mg/kg). In the acoustic startle and % PPI (***C***), no differences were found. Values significantly different from controls are indicated by asterisks (*p < 0.05, **p < 0.01; ***p < 0.001, Tukey’s or Sidak’s multiple comparisons tests). Values are expressed as mean ± SEM (for more details, see Extended Data Fig. 1-1). Download Figure 2-1, TIF file.

10.1523/ENEURO.0357-17.2018.f5-1 Extended Data Figure 5-1 Summary of results of the first cohort. In fear conditioning (***A***), BCI-838 caused reduced freezing in minute 2 of the contextual phase as well as the intertone and tone 2 periods of the cued phase. In the NO recognition (***B***), reduced exploration time was reversed by BCI-838. Values significantly different from controls are indicated by asterisks (*p < 0.05, **p < 0.01, ***p < 0.001, Tukey’s or Sidak’s multiple comparisons tests). Values are expressed as mean ± SEM (for more details, see Extended Data Fig. 1-1). Download Figure 5-1, TIF file.

### BCI-838 reverses chronic anxiety in blast-exposed rats

Starting at week 11 post-blast, rats were tested in an open field, an EZM, and a light/dark emergence task. No differences in the open field were found among the groups during the 60 min of testing (data not shown). In the light/dark emergence task (Fig. 2), blast-exposed rats treated with vehicle exhibited an increased latency to reach the light center (Fig. 2*B*; one-way ANOVA, *F*_(3,41)_ = 6.486, *p* = 0.0011), made fewer light center entries (Fig. 2*C*; *F*_(3,41)_ = 8.585, *p* = 0.0002), and traveled less distance on the light side (Fig. 2*E*; *F*_(3,42)_ = 8.547, *p* = 0.0002) compared to vehicle-treated controls. Treatment with high-dose BCI-838 (10 mg/kg/d) reversed deficits in the light center latency, light center entries and total distance traveled on the light side.

Twenty four h after the light/dark emergence task, rats were tested for 5 min in an EZM. Compared to vehicle-treated controls, blast-exposed rats treated with vehicle tended to moved less (Fig. 3*B*; *F*_(3,40)_ = 2.527, *p* = 0.071), showed an increased latency to reach an open arm (Fig. 3*C*; *F*_(3,40)_ = 5.080, *p* = 0.0045), made fewer open arm entries (Fig. 3*D*; *F*_(3,40)_ = 5.08, *p* < 0.0001), and spent less time in the open arms (Fig. 3*E*; *F*_(3,40)_ = 3.39, *p* = 0.0189). They also exhibited an increased latency to cross between two open arms (cross latency; Fig. 3*F*; *F*_(3,40)_ = 5.080, *p* = 0.0045). Treatment with 4 mg/kg and 10 mg/kg of BCI-838 reversed many of these effects. Results of treatment in cohort 1 revealed similar effects with BCI-838 reversing blast-associated anxiety in both light/dark emergence and the EZM (Extended Data Figs. 2-1*A*,*B*). Thus, blast-exposed rats exhibit signs of chronic anxiety in multiple tests that are reversed by treatment with BCI-838.

### Enhanced PPI in blast-exposed rats is unaltered with BCI-838 treatment

Enhanced acoustic startle is an important characteristic of the hyperarousal found in PTSD. Startle magnitude and sensory gating were examined in a PPI assay. Results of the first startle reactions are shown in Figure 4*A–C*. No differences were found between the groups whether vehicle or drug treated in background readings (pre; Fig. 4*A*; *F*_(3,39)_ = 2.398, *p* = 0.0826), acoustic startle response (pulse; Fig. 4*B*; *F*_(3,38)_ = 0.6367, *p* = 0.5960), or startle following the prepulse (Fig. 4*C*; *F*_(3,39)_ = 0.2161, *p* = 0.8846). An increased response was found between blast-exposed rats treated with vehicle and vehicle-treated controls when the first prepulse was subtracted from the first acoustic startle (pulse-prepulse; Fig. 4*D*). Blast-exposed rats treated with vehicle also exhibited an increased percentage of PPI versus vehicle-treated controls (Fig. 4*E*). Neither dose of BCI-838 affected startle magnitude or PPI among groups. Results were similar in the first cohort (Extended Data Fig. 2-1*C*). Thus, responses to auditory stimuli are altered following blast exposure but BCI-838 did not reverse these effects.

### Altered fear responses in blast-exposed rats are reversed with high-dose BCI-838

Models of conditioned fear are regarded as relevant to the study of the pathophysiological mechanisms of PTSD, where disordered fear regulation is observed (Mahan and Ressler, 2012). We examined blast-exposed rats in a cued/contextual fear paradigm (Fig. 5). Freezing behavior was measured during minutes 0–2 of the training session (baseline), after the presentation of the tone and after the footshock. Following the footshock, all groups showed increased freezing but no differences were found among groups (Fig. 5*A*; repeated-measures ANOVA, *F*_(2,42)_ = 401.01, *p* = 0.001 baseline vs post-shock and *F*_(2,42)_ = 0.211, *p* = 0.888 for freezing among groups by condition). On day 2 in the contextual phase, freezing was similar in all groups in minutes 1 and 2 (Fig. 5*B*). However, in minute 3, blast-exposed rats treated with low and high-dose BCI-838 showed less freezing compared to non-blast-exposed controls (one-way ANOVA, *F*_(3,43)_ = 2.857, *p* = 0.048 among groups min 3). On day 3, in the cued phase, blast-exposed rats treated with vehicle showed increased freezing in response to the second tone compared to vehicle-treated controls (Fig. 5*C*). Blast-exposed rats treated with low-dose BCI-838 showed less freezing compared with blast-exposed rats treated with vehicle in the second tone period (one-way ANOVA, *F*_(3,43)_ = 2.863, *p* = 0.0011 for freezing by condition tone 2). Moreover, blast-exposed rats treated with high-dose BCI-838 displayed less freezing compared with blast-exposed rats treated with vehicle in the intertone and tone 2 periods (one-way ANOVA, *F*_(3,43)_ = 103.84, *p* = 0.0001 among groups for intertone and *F*_(2,43)_ = 15.65, *p* = 0.0001 for tone 2).

In cohort 1, blast-exposed rats treated with vehicle showed a tendency to increased freezing in the cued phase o*f* testing compared to vehicle-treated controls (Extended Data Fig. 5-1*A*). Enhanced freezing during the cued phase of fear conditioning training has been observed in multiple other cohorts of rats studied in the past following a similar blast exposure protocol (Elder et al., 2012 and data not shown). Blast-exposed rats treated with high-dose BCI-838 displayed a tendency to less freezing compared with blast-exposed rats treated with vehicle in the intertone and tone 2 (Extended Data Fig. 5-1*A*). Thus, fear responses were chronically altered following blast exposure and reversed by BCI-838 4 and 10 mg/kg/d treatments in both of the cohorts.

### Altered NO recognition in blast-exposed rats is reversed with BCI-838

Cognitive impairment is a significant component of TBI and PTSD. As a measure of cognitive functioning in blast-exposed rats and the effects of BCI-838, we performed a NO recognition task. During the training phase, blast-exposed rats treated with vehicle explored the objects equally in each location but spent less total time in exploration (Fig. 6*E*) than all other groups (between-object discrimination comparisons were made using unpaired *t* tests (Student’s), *p* = 0.855 for discrimination Ob1 vs Ob2 controls; *p* = 0.7675 for blast exposed; *p* = 0.954 for blast exposed with BCI + LD and *p* = 0.724 for blast exposed with BCI + HD; Fig. 6*A*). When presented a NO, the vehicle-treated control and blast-exposed rats spent more time investigating the unfamiliar object, and the blast exposed again spent less total time in exploration (Fig. 6*E*) whether tested 1 h (STM; Fig. 6*B*; *p* < 0.0004 for discrimination FO vs NO controls; *p* < 0.0001 for discrimination FO vs NO blast exposed; *p* = 0.0002 for discrimination FO vs NO blast exposed with BCI +LD; and *p* < 0.0001 for discrimination FO vs NO blast-exposed BCI HD) or 24 h (LTM; Fig. 6*C*) after training (*p* < 0.0001 for discrimination FO vs NO controls; *p* < 0.0001 for discrimination FO vs NO blast exposed; *p* = 0.0017 for discrimination FO vs NO blast-exposed BCI LD and *p* < 0.0001 for discrimination FO vs NO blast-exposed BCI HD). Moreover, when an additional NO was presented four weeks after training (CM; Fig. 6*D*), blast-exposed rats treated with vehicle not only spent less time exploring both objects (familiar and novel) compared with non-blast-exposed controls (Fig. 6*E*), they also failed to explore the NO more than the familiar (*p* < 0.0083 for discrimination FO vs NO in controls; *p* = 0.5246 for discrimination FO vs NO blast exposed; *p* = 0.0003 for discrimination FO vs NO blast-exposed BCI LD and *p* = 0.0002 for discrimination FO vs NO blast-exposed BCI HD). Effects on reduced exploration time (Fig. 6*E*) as well as the late effects on CM (Fig. 6*D*) were reversed by low-dose (4 mg/Kg/d) and high-dose (10 mg/Kg/d) BCI-838. Results in cohort 1 were similar in STM and LTM testing (Extended Data Fig. 5-1*B*). Effects on CM at four weeks after training were not assessed in cohort 1.

### Enhanced neurogenesis in blast-exposed rats treated with BCI-838

**Figure 7.**
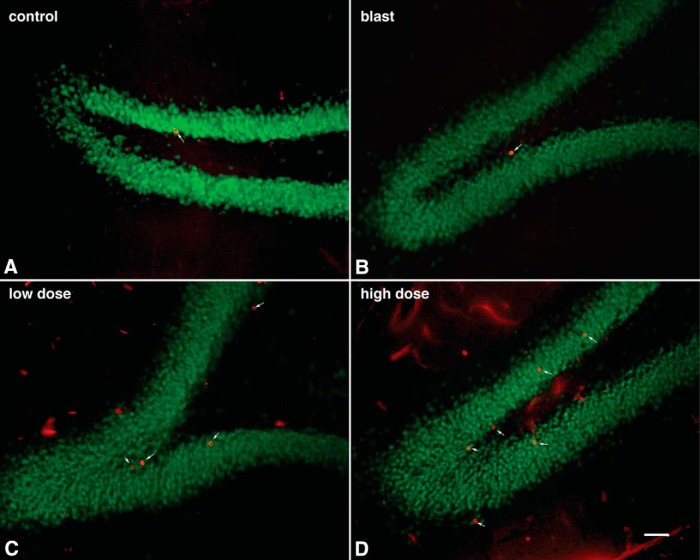
Neurogenesis is increased in blast-exposed rats stained for BrdU and the mature neuronal marker NeuN (BrdU-labeled cells are indicated with arrows). Representative confocal images (***A–D***) of the DG stained for BrdU (red) and the mature neuronal marker NeuN (green) in vehicle-treated controls (***A***), vehicle-treated blast-exposed (***B***) and blast exposed treated with low-dose (***C***) or high-dose (***D***) BCI-838. Scale bar: 50 μm.

mGluR2/3 receptor antagonists are known for their proneurogenic effects stimulating hippocampal neurogenesis in adult brain (Yoshimizu and Chaki, 2004). To determine the effects of blast and BCI-838 treatment on hippocampal neurogenesis, we first evaluated BrdU labeling of newly generated hippocampal cells at 10 weeks after the final BrdU injection (Fig. 7). We found no difference between numbers of BrdU-labeled cells in vehicle-treated blast-exposed and control rats suggesting that there is no inherent effect of blast on neural progenitor proliferation. However, we found a statistically significant increase in the number of BrdU-positive cells in blast-exposed rats treated with high-dose BCI-838 compared with blast exposed treated with vehicle suggesting that drug treatment induced neurogenesis in the hippocampus (Fig. 9*A*; one-way ANOVA, *F*_(3,11)_ = 3.446, *p* = 0.0401 blast exposed vs blast exposed treated with high-dose BCI and *p* = 0.0793 for treatment with low dose).

**Figure 8.**
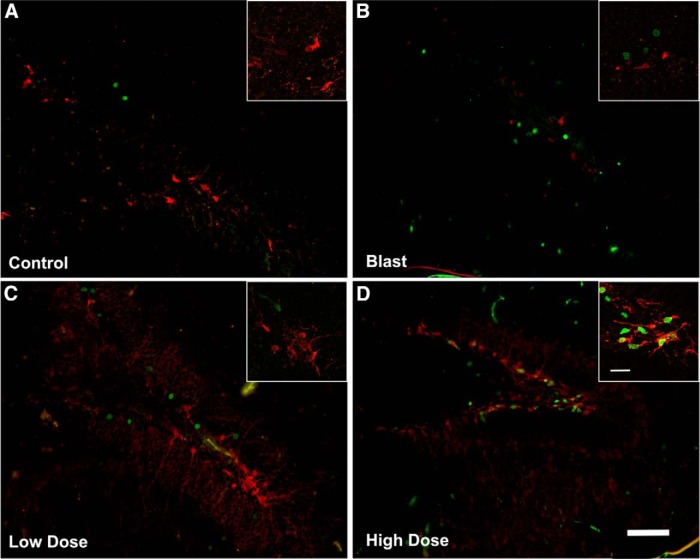
BCI-838 treatment increases the number of DCX-labeled cells in blast-exposed rats. Representative confocal images (***A–D***) of the DG stained for BrdU (green) and the young neuronal marker DCX (red) in vehicle-treated controls (***A***), vehicle-treated blast-exposed (***B***) and blast exposed treated with low-dose (***C***) or high-dose (***D***) BCI-838 (examples of DCX-labeled cells are indicated with arrows). Insets show examples of labeled cells viewed at higher power. We did not detect double-labeled cells stained with BrdU and DCX. Scale bar: 50 μm (panels) and 10 μm (insets).

**Figure 9.**
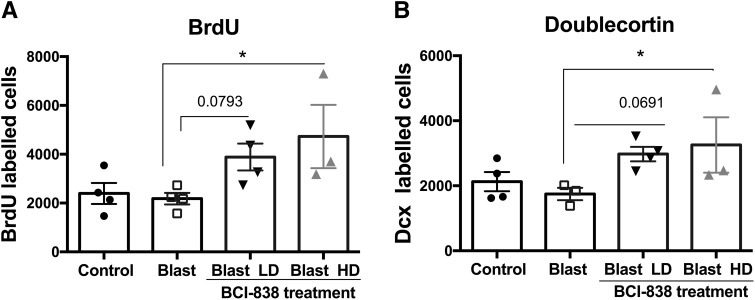
Quantification of neurogenesis in blast-exposed rats treated treated with vehicle or BCI-838. Data in ***A*** corresponds to the mean ± SEM of the total number of BrdU-labeled cells. Values significantly different from blast-exposed with vehicle and blast-exposed with drug are indicated by asterisks. Data in ***B*** corresponds to the mean ± SEM of the total number of DCX-labeled cells. Values significantly different from blast exposed treated with vehicle and blast exposed treated with drug are indicated by asterisks (*p < 0.05, ANOVA; Sidak’s multiple comparisons test).

However, given that BrdU labeling was examined 10 weeks after the last injection and not acutely, the analysis provided an estimation of cell survival, and the question remained as to whether BCI-838 treatment also stimulated cell proliferation. To assess this, a different set of slices from each experimental group was immunolabeled with antibodies against DCX and BrdU. In contrast to BrdU, which is a marker of cell proliferation since it is incorporated into DNA during the S-phase of the cell cycle, DCX is a microtubule-associated protein that in the adult brain labels immature neurons in the neurogenic niche (Brown et al., 2003) and is expressed specifically in virtually all migrating neuronal precursors of the developing CNS. In the adult hippocampus, DCX visualization gives a picture of the number of immature neurons. In agreement with the results of BrdU staining, blast-exposed rats treated with high-dose BCI-838 exhibited an increased number of DCX-labeled cells compared with blast exposed treated with vehicle (Figs. 8, 9*B* ). Thus, chronic treatment with BCI-838 does indeed increase hippocampal neurogenesis following blast injury.

## Discussion

TBI involves damage to the brain from an external force that can lead to direct tissue injury and hemorrhage as well as to the activation of secondary injury cascades that include inflammation and oxidative stress (Gennarelli and Grahm, 2005). TBI may also predispose to delayed neurodegeneration (DeKosky et al., 2010; Gandy et al., 2014; Elder, 2015). Postconcussion symptoms are often complicated by mental health problems including depression, anxiety and PTSD (Jorge et al., 2014). In particular, depression and PTSD have been common in former Warfighters returning from the recent conflicts in Iraq and Afghanistan (Hoge et al., 2008; Tanielian and Jaycox, 2008; Elder et al., 2010).

Animal models of blast-related TBI have studied the effects of differing blast pressures in the context of single or multiple exposures to determine how blast affects the nervous system and possible associations with mental health disorders including PTSD (Elder et al., 2010; Kobeissy et al., 2013; Elder et al., 2014). Here, we studied a model of blast exposure using rats that mimics a low-level blast exposure equivalent to a human mTBI or subclinical blast exposure (Ahlers et al., 2012; Elder et al., 2012). In rodents, studies have often documented transient behavioral changes following blast exposure but typically did not assess behavior beyond short-term acute effects (Kobeissy et al., 2013; Elder et al., 2014). Rats exposed to the protocol used here develop PTSD-related traits (including stress and generalized anxiety), that are still present many months after blast exposure suggesting that blast induces a chronic behavioral syndrome which may persist for the lifetime of the animal (Elder et al., 2012; Perez-Garcia et al., 2016, 2018). These traits include enhanced acoustic startle and anxiety in an EZM and light/dark emergence task. Rats also exhibit an enhanced cued fear conditioning response.

One striking feature of former Warfighters returning from the most recent conflicts has been the overlap of mTBI with PTSD (Elder et al., 2014). The presence of both disorders has complicated diagnosis, since clinically distinguishing a postconcussion syndrome from PTSD is often difficult. Indeed, some have suggested that blast-induced mTBI has been overdiagnosed (Hoge et al., 2009; Elder et al., 2014), with many of the symptoms being attributed to blast-related postconcussion syndrome better explained by PTSD (Hoge et al., 2009; Elder et al., 2014). However, it is intriguing that several case studies have noted that PTSD can develop following TBI in veterans who did not recall the traumatic experiences (Bryant, 2011). From the studies presented here as well as previous studies (Elder et al., 2012; Perez-Garcia et al., 2016, 2018), it appears that blast exposure per se can induce PTSD-like traits in blast-exposed rats without an added psychological stressor because blast exposures occurred under anesthesia. This is consistent with a series of former Warfighters in whom a novel occult astroglial scar at the junction of the cortical gray and white matter was recently identified as the structural basis for post-mTBI PTSD in a series of individuals (Shively et al., 2016).

The mechanism(s) underlying the development of PTSD-related behavioral traits after blast exposure remains unclear. In patients, neurobiological (neurochemical) and functional (neuroanatomical) abnormalities are commonly observed. Among the neurotransmitters in brain, amino acids like GABA and glutamate have a clear relationship to psychiatric disorders. Glutamate induces an excitatory synaptic signal and utilizes multiple receptors interacting and modulating cotransmitters in distinct regional brain areas associated with PTSD including the hippocampus, amygdala and cortex (Sherin and Nemeroff, 2011). mGluR2/3 receptors are distributed pre- and postsynaptically in neurons and are found on astrocytes as well. They modify the activities of other neurotransmitter systems such as dopamine and serotonin as well as affect glutamate signaling itself. Recently, selective mGluR2/3 receptors antagonists have been developed that increase synaptogenesis while at the same time modifying serotonergic and dopaminergic signaling (Chaki, 2017). Additionally, they exert antidepressant, anxiolytic, and procognitive effects in animal models.

Here, we show that BCI-838 can reverse multiple PTSD-related traits improving anxiety-related behaviors, fear responses, and long-term recognition memory. A major strength of the study is the replication of the effects of BCI-838 in two independent cohorts. A limitation of the study is the lack of inclusion of a sham-exposed control treated with drug although studies in mice have found that BCI-838 administration to wild type mice does not affect behavior (Kim et al., 2014). The fact that a mGluR2/3 antagonist, BCI-838, can reverse multiple PTSD-related traits in rats exposed to a blast overpressure injury while concomitantly enhancing neurogenesis in the DG indicates the involvement of glutamatergic components such as those in the hippocampus and cortex in the anxiety-related effects.

We evaluated primary anxiety in the light/dark emergence task (or light/dark box task), a test, which measures aversion to light and open spaces. In addition to aversion to light, blast-exposed rats made fewer entries and traveled less distance on the light side suggesting anxiety to novel and open spaces. The EZM is a measure of anxiety, which combines preference for closed versus open spaces with the added anxiety associated with elevation of the maze. It can also be interpreted as a cognitive assessment of risk test (Cryan and Sweeney, 2011). Blast-exposed rats moved less, made fewer open arm entries, and spent less time in the open arms as well as exhibited an increased latency to cross between two open arms (cross latency). Both light/dark emergence and EZM are based on the approach-avoidance conflict between stress (light, open space and/or elevation), and the natural exploratory tendency of rodents. In both, treatment with high-dose BCI-838 reversed (prevented) anxiety-related effects.

The acoustic startle reflex is a basic response to strong exteroceptive stimuli, and humans with PTSD show an enhanced response to acoustic startle (Orr et al., 1995; Morgan et al., 1996). While blast-exposed rats showed impaired PPI compared to non-blast-exposed controls neither dose of BCI-838 affected the abnormal responses to the pulse and prepulse. The inferior colliculus and intralaminar nucleus are critical parts of the auditory pathway mediating PPI of acoustic startle (Winer et al., 2002). Interestingly, neither structure has been reported to express mGluR2/3 receptors (Lu, 2014) suggesting a pharmacological explanation for the lack of BCI-838 effect.

In contrast, metabotropic glutamate receptor distribution in hippocampus and amygdala is dense and essential for consolidation and extinction of fear conditioning in rodents. Individuals with PTSD typically show increased sensitization to stress, overgeneralization of fear to irrelevant stimuli, and impaired extinction of fear memories (Mahan and Ressler, 2012). Fear responses were attenuated by both the 4- and 10-mg/kg treatment doses of BCI-838 suggesting participation of glutamatergic components in the hippocampus and cortex (Higgins et al., 2004; Li et al., 2011; Popoli et al., 2011) and in the hippocampus and amygdala which are essential for consolidation and extinction of fear conditioning in rodents. Indeed, as shown in Figure 5, all groups in cohort 2 responded with similar freezing during the first min of the contextual phase indicating that they remembered and initially responded to the previously encountered context with a similar response. However, in minute 3, both drug-treated groups froze less arguing that the main drug effect was not on fear memory but on how the fear response was sustained. A similar conclusion can be drawn from the cued phase testing (Fig. 5*C*) in which group differences were not seen in freezing to the initial tone but rather in the intertone and second tone periods where rats treated with high-dose BCI-838 froze less. Similar trends were seen in cohort 1 (Extended Data Fig. 5-1) reflected in less freezing in drug-treated rats in the third min of the contextual phase and in the intertone and tone 2 intervals in the cued phase. Collectively these results suggest that BCI-838 does not directly affect fear memory but rather produces an habituation effect on the fear response.

Cognitive problems including deficits in attention, learning, and memory are common in former Warfighters following blast injury (Elder et al., 2010). NO recognition is a task dependent on extra hippocampal regions that have a dense population of glutamatergic receptors. When blast-exposed rats were tested in a NO recognition task, they spent less total time exploring whatever objects were presented during training, STM (1 h) and LTM (24 h) testing. However, as did controls, blast-exposed rats discriminated the novel from the FO and spent more time exploring the NO during the STM and LTM testing. Treatment with low and high doses of BCI-838 reversed lowered exploration time, particularly the 10 mg/kg dose, which restored exploration time to the same magnitude as controls. When we conducted delayed testing four weeks after initial training (and five months post-blast exposure), blast-exposed rats explored the NO no more than the FO, while controls retained the memory of the previously FO and explored the NO more. Treatment with low and high doses of BCI-838 prevented this amnesic effect.

How BCI-838 exerts it beneficial effects on the behavioral changes that follow blast injury in rats remains incompletely understood. mGluR2/3 receptor antagonists are known for their proneurogenic effects, stimulating hippocampal neurogenesis in adult brain (Yoshimizu and Chaki, 2004). Our studies herein provide the first quantitative demonstration of increased neurogenesis in the DG following chronic BCI-838 administration in an animal model of blast-related TBI. We observed a significant increase of proliferating cells (BrdU-positive) and an increase of immature neurons (DCX-positive) in BCI-838-treated animals. Other work has shown that the number of DCX-expressing cells correlates with the level of cellular proliferation in the DG (Brown et al., 2003; Rao and Shetty, 2004). These results demonstrate that chronic BCI-838 administration to blast-exposed rats is associated with increased DG-cell proliferation, robustly increasing numbers of immature neurons, which appear to remain in a less differentiated state. Thus, increased neurogenesis could be one mechanism whereby BCI-838 rescues the chronic PTSD-related behavioral phenotype despite the fact that blast-exposed rats exhibited no deficit per se in neurogenesis. However, the roles of mGluR 2/3 receptors in neurons and glial cells are not fully known and receptor blockade by BCI-838 may also exert neuroprotective actions through other mechanisms that aid in reversal of the phenotype.

Regardless of whatever the mechanism of action, we show that BCI-838 is a promising drug to reverse PTSD-related traits in a rat model of mTBI improving anxiety-related behaviors, fear responses, and long-term recognition memory in blast-exposed rats. Although BCI-838 increased hippocampal neurogenesis in blast-exposed rats, this drug could affect the glutamatergic system in other ways that contribute to its efficacy in treating PTSD-related traits. As with refractory major depression and suicidality, current therapies are only partially effective for treatment of PTSD-related symptoms following blast injury. The present study highlights BCI-838, hippocampal neurogenesis, and the mGluR2/3 pathway as potential leads in the development of novel pharmacological therapies for former Warfighters suffering from PTSD symptoms. The blast protocol described here also provides a model to study the chronic and persistent behavioral effects of blast including the relationship between PTSD and mTBI in dual diagnosis former Warfighters and a model to test new therapeutic strategies to relieve the PTSD symptoms in this population.

## Synthesis

Reviewing Editor: Jeffery Twiss, University of South Carolina Decisions are customarily a result of the Reviewing Editor and the peer reviewers coming together and discussing their recommendations until a consensus is reached. When revisions are invited, a fact-based synthesis statement explaining their decision and outlining what is needed to prepare a revision will be listed below. The following reviewer(s) agreed to reveal their identity: C. Edward Dixon. Note: If this manuscript was transferred from JNeurosci and a decision was made to accept the manuscript without peer review, a brief statement to this effect will instead be what is listed below.

Post-traumatic stress disorder (PTSD) is a frequent mental health problem of soldiers returning from recent conflicts and is manifested by cognitive impairment, depression, fear response, sleep disorders, etc. The present study demonstrates that pharmacological treatment with mGluR2/3 receptor antagonist (BCL-838) can modify or reverse the PTSD-related behaviors in a rat blast injury model. Both reviewers and I agree that you show impressive beneficial effects from the drug in behavior analyses and hippocampal neurogenesis. The strengths of the study include the use of a comprehensive battery of behavior tests, the use of a clinically relevant dosing regimen, and that no drug was onboard during behavioral testing. The manuscript provides a clue to the mechanism(s) that may underlie these effects, at least in part, by showing increased levels of proliferating cells (BrdU positive) and immature neurons (DCX-positive) in the dentate gyrus. Overall, the data provide a translational advance for understanding the effects of BCL-838. Together, we think the following concerns need to be addressed in a revised manuscript:

1. The rationale for two cohorts is not obvious. It appears that repeating the behavioral testing in the animals with neurogenesis outcomes proved that the two cohorts were equivalently injured. Since the beneficial effects of BCI-838 were seen in two cohorts, the fact that efficacy was replicated should be highlighted in the Discussion.

2. The data for second cohort of animals largely show validation of the results. Consequently, Figures 10 and 11 do not add much beyond Figures 2-4 and 5-6, respectively. So these Figures should be moved to extended data. Please note that the y-axis in Fig 3B is 10-fold that of the corresponding graph in Figure 10B and should be edited.

3. There are sufficient statistical details in the Results and Methods sections so the cohort data in Tables 1 and 2 should be moved to extended data.

4. There is a literature on metabotrophic receptor targeted therapies in experimental traumatic brain injury that should be mentioned in the Introduction.

5. Absence of a sham plus drug group should be included in the Discussion as a limitation of the study.

6. Interpreting fear response data is always challenging, so alternative interpretations should be considered. For example, does a reduction in freezing mean less fear or less learning?

7. The authors need to clarify if body temperature was monitored before and after the blast exposure.

8. Objects used for the novel object recognition test should be included in the methods section.

## Author Response

Dear Dr. Twiss,

Thank you for your willingness to consider a revised version of our paper “PTSD-related behavioral traits in a rat model of blast-induced mTBI are reversed by the proneurogenic mGluR2/3 receptor antagonist BCI-838” previously submitted to eNeuro. Specific revisions in response to the reviewer's comments are described below. The reviewer comments are marked in italics. Changes to the manuscript are marked in red in the text.

Significance Statement Comments for Author (Required): Could be shortened without losing the pertinent significance.

The significance statement has been shortened from 108 words to 61 words.

“1. The rationale for two cohorts is not obvious. It appears that repeating the behavioral testing in the animals with neurogenesis outcomes proved that the two cohorts were equivalently injured. Since the beneficial effects of BCI-838 were seen in two cohorts, the fact that efficacy was replicated should be highlighted in the Discussion”.

We can appreciate that the rationale for the two cohorts was anything but obvious. To be confident of an adequately powered study, we originally planned on having four groups of rats at 20/group (i.e. 80 rats total for the study). Due to the size of the study we decided to split it into two separate experiments with the intention of combining the data from the two cohorts using z-scores. Unfortunately due to unexpected animal death we ended up with unbalanced groups making it difficult to combine the two data sets using z-scores. However, we found that when we analyzed the two cohorts separately, despite the relatively modest group sizes, the effect sizes were large enough to see differences that were largely replicated between the two cohorts. We therefore decided to present the two cohorts separately in the paper. The fact that efficacy was replicated in both groups is now highlighted in the “Discussion” on page 25.

2. The data for second cohort of animals largely show validation of the results. Consequently, Figures 10 and 11 do not add much beyond Figures 2-4 and 5-6, respectively. So these Figures should be moved to extended data. Please note that the y-axis in Fig 3B is 10-fold that of the corresponding graph in Figure 10B and should be edited.

As suggested we have moved figures 10 and 11 to the extended data section (now labeled figures 1 and 2). We thank the reviewers for pointing out the problem with the Y-axis in Fig 3B. This has been corrected.

3. There are sufficient statistical details in the Results and Methods sections so the cohort data in Tables 1 and 2 should be moved to extended data.

As suggested we have moved Tables 1 and 2 to the extended data section.

4. There is a literature on metabotrophic receptor targeted therapies in experimental traumatic brain injury that should be mentioned in the Introduction.

We thank the reviewers for pointing out the prior literature on metabotropic receptor treatments in TBI. These are now cited in the “Introduction” on page 5

5. Absence of a sham plus drug group should be included in the Discussion as a limitation of the study.

A sham group treated with drug was not included out of concerns over expanding the already large size of the study. We also knew from studies in mice that BCI-838 administration to wild type mice did not seem to affect behavior. However, we agree that the lack of inclusion of a sham plus drug treated group is a limitation of the study and this is now acknowledged in the discussion on page 25.

6. Interpreting fear response data is always challenging, so alternative interpretations should be considered. For example, does a reduction in freezing mean less fear or less learning?

We agree that interpreting fear-conditioning data is often challenging. Indeed the reviewers raise an interesting question that perhaps we had not considered as much as we might as to whether the BCI-838 effect is more on fear learning or the fear response itself. In fact, if one examines figure 5, all groups in cohort 2 responded with similar freezing during the first min of the contextual phase suggesting that all groups remembered and initially responded to the previously encountered context with a similar response. What differed was that at 3 min, both drug treated groups froze less arguing that the main effect of the drug was not on fear learning or memory but on how sustained the fear response was. A similar conclusion can be drawn for the cued phase testing (Fig. 5C) where group differences were not in freezing to the initial tone but rather in the intertone and second tone periods where rats treated with high dose BCI-838 froze less. Similar trends were seen in cohort 1 (Extended Data figure 2 in the revised manuscript) in that where trends were seen they were reflected in less freezing in drug treated rats in the third min of the contextual phase and in the intertone and tone 2 intervals in the cued phase. We thank the reviewers for raising this issue and have revised both the description of the fear testing results on page 19 and the “Discussion” on page 26 to reflect these observations.

7. The authors need to clarify if body temperature was monitored before and after the blast exposure.

We did not measure body temperature before and after blast exposure. However, the length of time under anesthesia including placement in the shock tube and execution of the blast procedure is less than 3 minutes and under the blast conditions utilized here blast-exposed rats recovery identically to controls and exhibit no loss of the righting reflex. Thus while we cannot exclude that a reduction in core temperature occurs any reduction would not appear to affect recovery. On page 8 of the revised manuscript in the “Materials and Methods” subsection “Blast overpressure exposure” we have added statements regarding the length of time under anesthesia and the similar recovery patterns of the blast exposed.

8. Objects used for the novel object recognition test should be included in the methods section.

The objects used were a 330 ml soda can, a metal box, a cup and a plastic tube. They were selected to be of similar size, but different in shape and color and were made of ceramic or metal material. The specific objects used have been added to the “Methods” section on page 12.

Thank you again for the opportunity to respond to the comments and criticisms of the reviewers. We believe that the careful review given our manuscript has significantly improved both its content and clarity. We hope that the revised manuscript will be of interest to readers of eNeuro. Please feel free to contact us if we can provide any additional information or if you require any additional modifications or clarifications.
