# Supplementary material for: PTSD-Related Behavioral Traits in a Rat Model of Blast-Induced mTBI Are Reversed by the mGluR2/3 Receptor Antagonist BCI-838
Source: eNeuro. 2018 Jan 30;5(1):ENEURO.0357-17.2018. doi: 10.1523/ENEURO.0357-17.2018 (PMC5790754; doi:10.1523/ENEURO.0357-17.2018)
Supplement: Extended Data Figure 1-1 — Details of statistical analysis cohort one. Download Figure 1-1, DOCX file. [file sup_enu-eN-NWR-0357-17-s01.docx]

**Figure 1-1. Details of statistical analysis cohort one.**

| Figure  (Extended Data) | Data structure | Type of test | Observed  power | n |
| --- | --- | --- | --- | --- |
| 2-1A  (LD) | Nonparametric | One-Way ANOVA/Sidak’s multiple comparison | 0.72 | Control 7  Blast 7  Blast LD 7  Blast HD 5 |
| 2-1A | Normal distribution | One-Way ANOVA/Sidak’s multiple comparison | 0.85 | Control 7  Blast 7  Blast LD 7  Blast HD 5 |
| 2-1A | Normal distribution | One-Way ANOVA/Sidak’s multiple comparison | 0.80 | Control 7  Blast 7  Blast LD 6  Blast HD 5 |
| 2-1A | Normal distribution | One-Way ANOVA/Sidak’s multiple comparison | 0.99 | Control 7  Blast 7  Blast LD 7  Blast HD 5 |
| 2-1A | Normal distribution | One-Way ANOVA/Sidak’s multiple comparison | 0.80 | Control 7  Blast 7  Blast LD 7  Blast HD 5 |
| 2-1B  (Zero Maze) | Normal distribution | One-way ANOVA/Tukey's multiple comparisons test | NA | Control 7  Blast 7  Blast LD 7  Blast HD 5 |
| 2-1B | Normal distribution | One-Way ANOVA/Sidak’s multiple comparison | 0.99 | Control 7  Blast 7  Blast LD 7  Blast HD 5 |
| 2-1B | Normal distribution | One-Way ANOVA/Sidak’s multiple comparison | 0.93 | Control 7  Blast 7  Blast LD 7  Blast HD 5 |
| 2-1B | Normal distribution | One-Way ANOVA/Sidak’s multiple comparison | 0.93 | Control 7  Blast 7  Blast LD 7  Blast HD 5 |
| 2-1B | Normal distribution | One-Way ANOVA/Sidak’s multiple comparison | 0.799 | Control 7  Blast 7  Blast LD 7  Blast HD 5 |
| 2-1C  (SPI) | Normal distribution | One-way ANOVA /Tukey's multiple comparisons test | 0.2 | Control 7  Blast 7  Blast LD 7  Blast HD 5 |
| 2-1C | Normal distribution | One-way ANOVA /Tukey's multiple comparisons test | 0.5 | Control 7  Blast 7  Blast LD 7  Blast HD 5 |
| 2-1C | Normal distribution | One-way ANOVA /Tukey's multiple comparisons test | 0.2 | Control 6  Blast 6  Blast LD 7  Blast HD 5 |
| 2-1C | Normal distribution | One-way ANOVA /Tukey's multiple comparisons test | 0.2 | Control 6  Blast 6  Blast LD 7  Blast HD 5 |
| 5-1A (FC) | Normal distribution | Repeated measures | NA | Control 6  Blast 6  Blast LD 7  Blast HD 5 |
| 5-1A | Normal distribution | One-way ANOVA  Sidak's multiple comparisons test | 0.81 for last minute | Control 6  Blast 6  Blast LD 7  Blast HD 5 |
| 5-1A | Normal distribution | One-way ANOVA  Sidak's multiple comparisons test | 0.87 for tone 2 | Control 6  Blast 6  Blast LD 7  Blast HD 5 |
| 5-1B (NOR) | Normal distribution | unpaired *t*-tests | 0.7 for controls | Control 7  Blast 6  Blast LD 7  Blast HD 5 |
| 5-1B | Normal distribution | unpaired *t*-tests | 0.98 for controls | Control 7  Blast 6  Blast LD 7  Blast HD 5 |
| 5-1B | Normal distribution | unpaired *t*-tests | 0.99 for controls | Control 7  Blast 6  Blast LD 7  Blast HD 5 |
| 5-1B | Normal distribution | One-Way ANOVA/Sidak’s multiple comparison | 0.98 | Control 7  Blast 7  Blast LD 7  Blast HD 5 |
