# Supplementary material for: PTSD-Related Behavioral Traits in a Rat Model of Blast-Induced mTBI Are Reversed by the mGluR2/3 Receptor Antagonist BCI-838
Source: eNeuro. 2018 Jan 30;5(1):ENEURO.0357-17.2018. doi: 10.1523/ENEURO.0357-17.2018 (PMC5790754; doi:10.1523/ENEURO.0357-17.2018)
Supplement: Extended Data Figure 1-2 — Details of statistical analysis cohort two. Download Figure 1-2, DOCX file. [file sup_enu-eN-NWR-0357-17-s02.docx]

**Figure 1-2. Details of statistical analysis cohort two.**

| Figure | Data structure | Type of test | Observed power | n |
| --- | --- | --- | --- | --- |
| 2A | Normal distribution | One-way ANOVA/Tukey's multiple comparisons test | 0.63 | Control 8  Blast 13  Blast LD 13  Blast HD 12 |
| 2B | Normal distribution | One-way ANOVA/Tukey's multiple comparisons test | 0.973 | Control 8  Blast 12  Blast LD 13  Blast HD 11 |
| 2C | Normal distribution | One-way ANOVA/Tukey's multiple comparisons test | 0.986 | Control 8  Blast 13  Blast LD 13  Blast HD 12 |
| 2D | Normal distribution | One-Way ANOVA/Sidak’s multiple  comparisons test | 0.9896 | Control 8  Blast 13  Blast LD 13  Blast HD 12 |
| 2E | Normal distribution | One-way ANOVA/Tukey's multiple comparisons test | 0.9878 | Control 8  Blast 13  Blast LD 13  Blast HD 12 |
| 3A | Normal distribution | One-way ANOVA/Tukey's multiple comparisons test | 0.132 | Control 8  Blast 13  Blast LD 13  Blast HD 11 |
| 3B | Normal distribution | One-Way ANOVA/Sidak’s multiple comparison | 0.610 | Control 8  Blast 13  Blast LD 12  Blast HD 11 |
| 3C | Normal distribution | One-way ANOVA/Tukey's multiple comparisons test | 0.9608 | Control 8  Blast 13  Blast LD 12  Blast HD 11 |
| 3D | Normal distribution | One-way ANOVA/Tukey's multiple comparisons test | 0.9012 | Control 8  Blast 12  Blast LD 12  Blast HD 11 |
| 3E | Normal distribution | One-Way ANOVA/Sidak’s multiple comparison | 0.9050 | Control 8  Blast 13  Blast LD 12  Blast HD 11 |
| 3F | Normal distribution | One-Way ANOVA/Sidak’s multiple comparison | 0.898 | Control 8  Blast 13  Blast LD 12  Blast HD 11 |
| 4A | Normal distribution | One-way ANOVA/Tukey's multiple comparisons test | 0.99 | Control 8  Blast 12  Blast LD 11  Blast HD 12 |
| 4B | Normal distribution | One-way ANOVA /Tukey's multiple comparisons test | 0.675 | Control 8  Blast 12  Blast LD 11  Blast HD 12 |
| 4C | Normal distribution | One-way ANOVA /Tukey's multiple comparisons test | 0.99 | Control 8  Blast 12  Blast LD 11  Blast HD 12 |
| 4D | Normal distribution | One-way ANOVA /Tukey's multiple comparisons test | 0.99 | Control 8  Blast 12  Blast LD 11  Blast HD 12 |
| 4E | Normal distribution | One-way ANOVA /Tukey's multiple comparisons test | 0.98 | Control 7  Blast 10  Blast LD 10  Blast HD 12 |
| 5A | Normal distribution | Repeated measures | NA | Control 9  Blast 13  Blast LD 13  Blast HD 12 |
| 5B | Normal distribution | One-way ANOVA  Sidak's multiple comparisons test | 0.99 for last min | Control 9  Blast 13  Blast LD 13  Blast HD 12 |
| 5C | Normal distribution | One-way ANOVA/  Tukey's multiple comparisons test | 0.985 for tone 2 | Control 9  Blast 11  Blast LD 13  Blast HD 12 |
| 6A | Normal distribution | unpaired *t*-tests | 0.980 for controls | Control 9  Blast 13  Blast LD 13  Blast HD 12 |
| 6B | Normal distribution | unpaired *t*-tests | 0.969 for controls | Control 9  Blast 13  Blast LD 13  Blast HD 12 |
| 6C | Normal distribution | unpaired *t*-tests | 0.968 for controls | Control 9  Blast 13  Blast LD 13  Blast HD 12 |
| 6D | Normal distribution | unpaired *t*-tests | 0.95 for controls | Control 8  Blast 13  Blast LD 12  Blast HD 11 |
| 6E | Normal distribution | One-way ANOVA/  Tukey's multiple  comparison | 0.99 | Control 8  Blast 13  Blast LD 12  Blast HD 11 |
| 9A | Normal distribution | One-Way ANOVA/Sidak’s multiple comparison | 0.99 | Control 4  Blast 3  Blast LD 4  Blast HD 3 |
| 9B | Normal distribution | One-Way ANOVA/Sidak’s multiple comparison | 0.90 | Control 4  Blast 4  Blast LD 4  Blast HD 3 |
