# Supplementary figures and images for: PTSD-Related Behavioral Traits in a Rat Model of Blast-Induced mTBI Are Reversed by the mGluR2/3 Receptor Antagonist BCI-838
Source: eNeuro. 2018 Jan 30;5(1):ENEURO.0357-17.2018. doi: 10.1523/ENEURO.0357-17.2018 (PMC5790754; doi:10.1523/ENEURO.0357-17.2018)

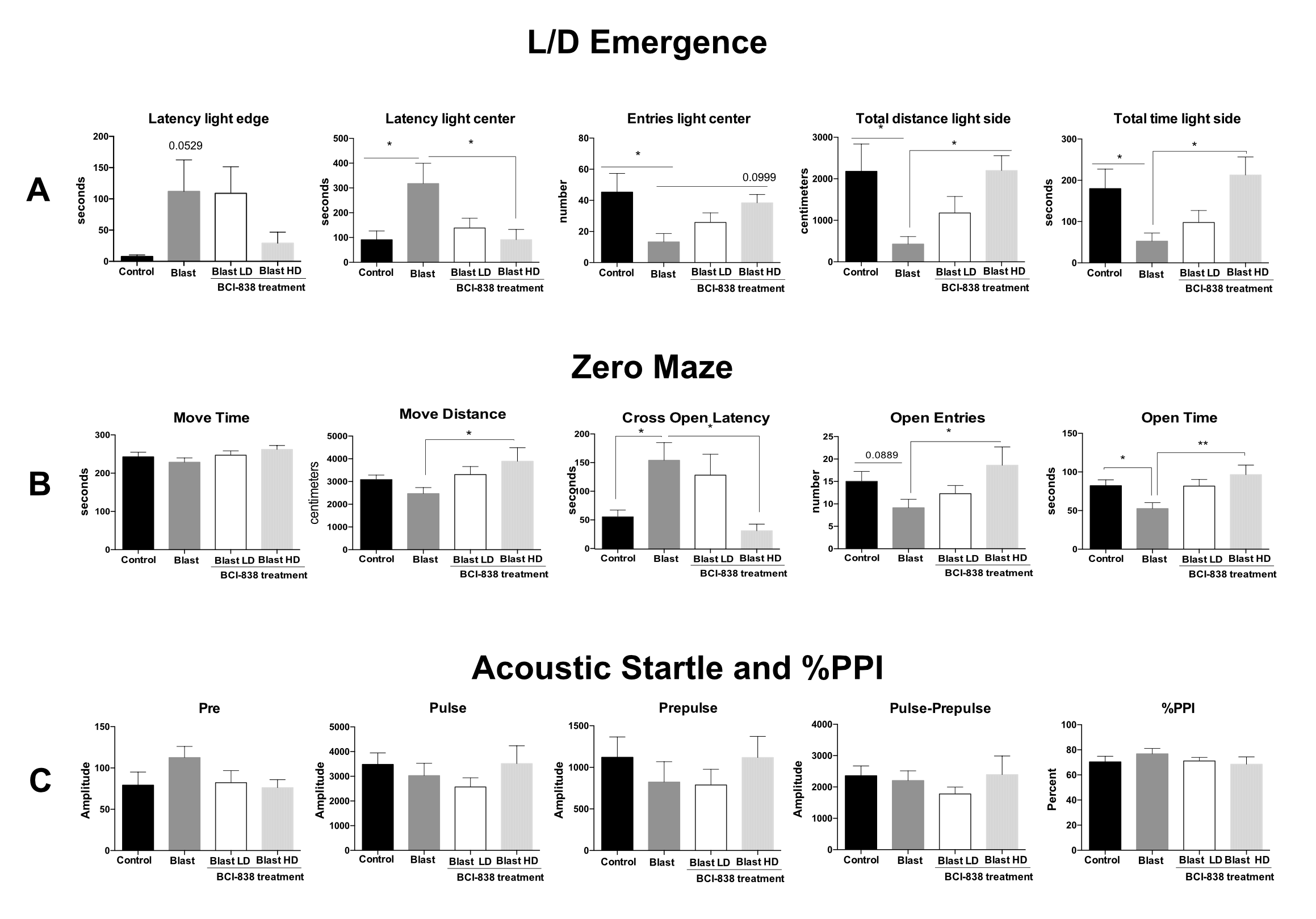

Supplement: Extended Data Figure 2-1 — Summary of results of the first cohort. High-dose BCI-838 reverses anxiety. In the light/dark emergence task (A), the blast-exposed rats exhibited an increased latency to reach the light center, made fewer light center entries, traveled less distance, and spent less time on the light side compared to vehicle-treated controls, effects that were mostly reversed by high-dose BCI-838. In the zero maze (B), blast-exposed rats showed an increased open arm cross latency, tended to make fewer open entries, and spent less time in the open arms. These parameters were reversed by BCI-838 (10 mg/kg). In the acoustic startle and % PPI (C), no differences were found. Values significantly different from controls are indicated by asterisks (*p < 0.05, **p < 0.01; ***p < 0.001, Tukey’s or Sidak’s multiple comparisons tests). Values are expressed as mean ± SEM (for more details, see Extended Data Fig. 1-1). Download Figure 2-1, TIF file. [file sup_enu-eN-NWR-0357-17-s03.tif]

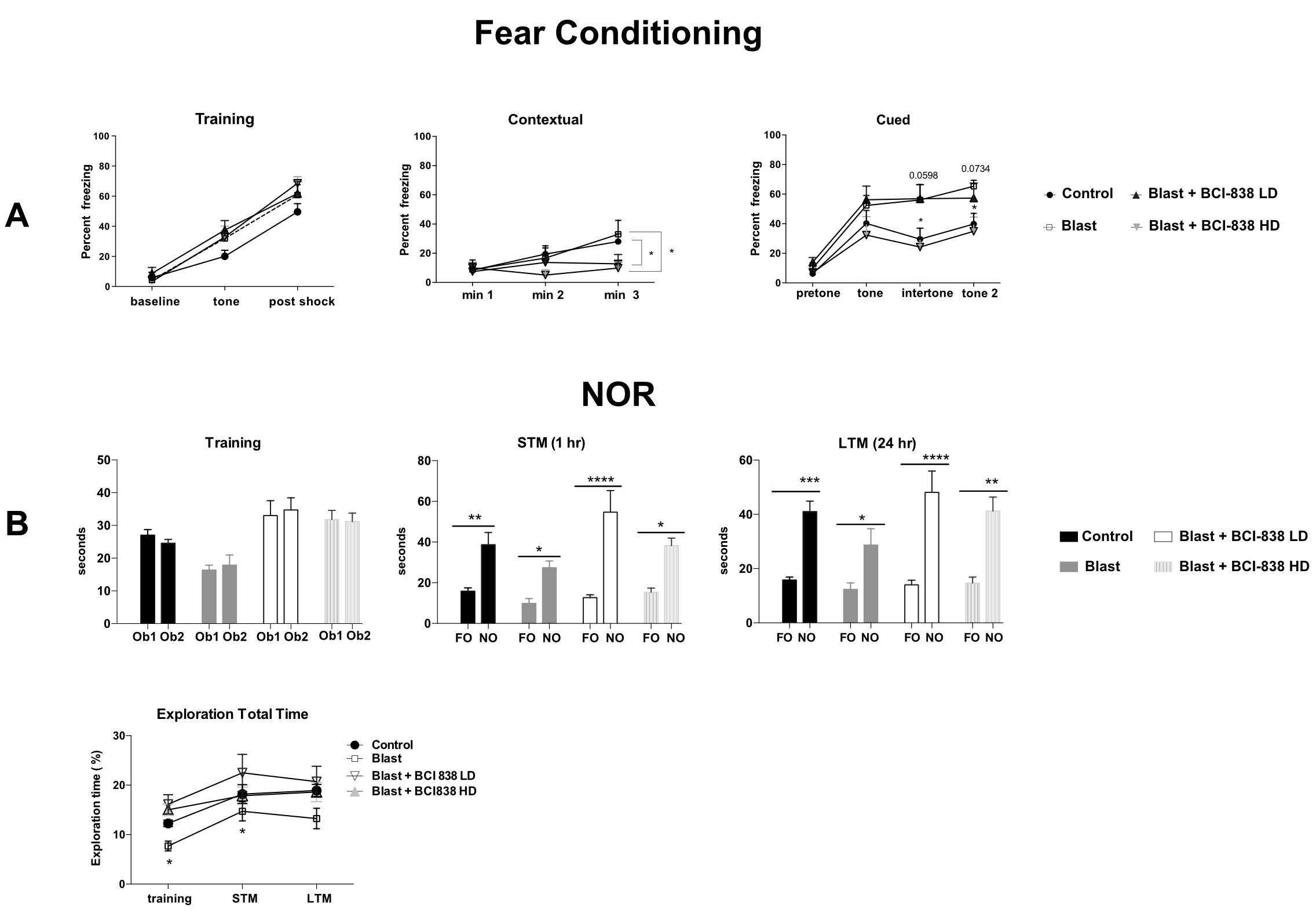

Supplement: Extended Data Figure 5-1 — Summary of results of the first cohort. In fear conditioning (A), BCI-838 caused reduced freezing in minute 2 of the contextual phase as well as the intertone and tone 2 periods of the cued phase. In the NO recognition (B), reduced exploration time was reversed by BCI-838. Values significantly different from controls are indicated by asterisks (*p < 0.05, **p < 0.01, ***p < 0.001, Tukey’s or Sidak’s multiple comparisons tests). Values are expressed as mean ± SEM (for more details, see Extended Data Fig. 1-1). Download Figure 5-1, TIF file. [file sup_enu-eN-NWR-0357-17-s04.tif]
